# Electro-optic frequency shift of single photons from a quantum dot

**DOI:** 10.1515/nanoph-2024-0550

**Published:** 2025-01-21

**Authors:** Sanjay Kapoor, Aleksander Rodek, Michał Mikołajczyk, Jerzy Szuniewicz, Filip Sośnicki, Tomasz Kazimierczuk, Piotr Kossacki, Michał Karpiński

**Affiliations:** Faculty of Physics, 49605 University of Warsaw, Warsaw, Poland; Integrated Quantum Optics, Institute for Photonic Quantum Systems (PhoQS), Paderborn University, Paderborn, Germany

**Keywords:** quantum dots, single-photon source, electro-optic phase modulation, quantum photonics, quantum interface, spectral shift

## Abstract

Quantum dots (QDs) are a promising source of single photons mainly due to their on-demand operation. However, their emission wavelength depends on their size and immediate surroundings in the solid-state environment. By applying a serrodyne electro-optic phase modulation, we achieve a spectral shift up to 0.01 nm (3.5 GHz) while preserving the purity and indistinguishability of the photons. This method provides an efficient and scalable approach for tuning the emission wavelength of QDs without relying on nonlinear frequency mixing or probabilistic processes. Our results show that the electro-optic phase modulation enables stable and tunable spectral shifts, making it suitable for applications such as quantum communication, quantum key distribution, and primarily integrating remote quantum dot sources into large-scale quantum networks.

## 1 Introduction

There is a growing interest in producing indistinguishable multiphoton states for advanced quantum optics experiments [1], [2], [3], [4], including boson sampling [5], [6], and large-scale quantum information processing and distribution. In recent years, semiconductor quantum dots (QDs) have emerged as an attractive candidate for sources of multiphoton states due to their on-demand operation, high brightness, purity, and indistinguishability [7], [8], while retaining compatibility with various photonic integrated platforms [9], [10], [11], [12]. However, due to imperfections in fabrication processes, QDs have variations in structural properties, which lead to different emission wavelengths [13], [14], [15].

To generate multiple indistinguishable photons from distinct QDs, it is essential to match both the emission wavelengths and the spectro-temporal waveforms of the emitted photons [16], [17], [18]. In a broader context, the ability to control the central wavelength and spectro-temporal waveforms is crucial for interfacing various quantum information processing units into a large-scale quantum network and for high-dimensional quantum information encoding [19], [20].

The emission from QDs can be tuned either by modifying their physical properties during their growth or by changing various parameters of the QD environment. Examples include quantum-confined Stark/Zeeman effects — adjusting the emission energy levels through an external electric/magnetic field [21], [22], applying mechanical strain to modify the QD’s emission properties [23], [24], or altering the QD’s temperature to shift its emission wavelength [25], [26], [27]. These techniques, while effective, are challenging to scale and generally require advanced sample processing adding to the cost and complexity of any potential device. Alternatively, QD photons can be optically manipulated after their emission.

The optical techniques for frequency shifting and spectro-temporal waveform shaping of heralded single photons from a parametric down-conversion source have been studied extensively [20], [28], [29], [30], [31], [32], [33], [34], [35], [36]. However, there are limited works involving photons emitted from QD sources. Frequency shifting of QD photons has been realized by nonlinear optical frequency mixing [37], [38], [39]. This approach provides large spectral shifts, but the conversion is probabilistic, i.e., there can be unconverted photons at the output depending on the conversion efficiency of the nonlinear process, and the process is strictly phase matching dependent, which limits the fine-tuning around the translated wavelength. Electro-optic intensity modulation of QD photons has been used to shape their temporal waveform [40], where the challenge of synchronizing the electro-optic waveform to the single-photon pulses is discussed. Temporal shaping of QD photons has also been performed by an electro-optically modified pump [41]. Electro-optic phase modulation has been used to generate spectral sidebands of QD photons [42]; however, this technique is inefficient for frequency shifting because it redistributes the photon’s spectrum in multiple blue-and-red shifted sidebands.

In this work, we use a fast electro-optic phase modulator (EOPM) to implement a serrodyne frequency translator [43]. Our setup shifts the central frequency of the QD photons by 0.01 nm (3.5 GHz), while preserving the purity and indistinguishability of the source. The internal electro-optic conversion efficiency is equal to unity, with every output photon undergoing the same spectral transformation. As a single-photon source, we use a commercially available photonic integrated chip from Sparrow Quantum. The chip contains InAs QDs embedded in a photonic crystal waveguide. The QD emits in the wavelength range of 920–940 nm. Our approach is adaptable to a variety of QDs, as phase modulators are commercially available for a range of wavelength regions spanning from visible (400 nm) to mid-infrared (2,000 nm), thanks to the wide transparency window of lithium niobate, a common electro-optic material [44], which is employed in this work.

The manuscript is organized as follows: In Section 2, we describe the experimental setup and introduce the concept of electro-optic spectral shift. We also discuss the measurement schemes used for characterizing the single-photon source with shifted and unshifted photons. In Section 3, we present and discuss the results. Finally, in the last section, we provide our conclusions and discuss future prospects.

## 2 Experimental setup and methods

**Figure 1:**
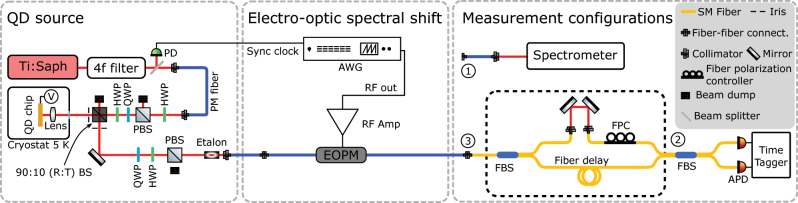
Schematic of the experimental setup: The QD is resonantly excited using a pulsed ps Ti:Sapphire laser, and the QD emission is collected into a polarization maintaining fiber (PM fiber). The collected emission is passed through a fiber-coupled phase modulator (EOPM), which is driven by a sawtooth RF waveform implementing a serrodyne frequency shift. For spectral measurements, the output fiber end of the EOPM is connected to a spectrometer at port (1). For single-photon purity measurements, the output of the EOPM is connected at port (2), skipping the interferometer setup (dashed-black box). For photon indistinguishability measurements, the output is connected at port (3) to an unbalanced in-fiber Mach–Zehnder interferometer, with a 26 ns delay. The EOPM acts passively when no voltage is applied, allowing comparison between shifted and unshifted photons.

This section outlines the detailed configuration of our experimental setup as depicted in Figure 1, and the methodology used to demonstrate the electro-optic spectral shift of single photons from a QD source. The setup is divided into three key parts: the single-photon source, the electro-optic frequency shift, and the measurement schemes.

### 2.1 Quantum dot single-photon source

The single-photon source consists of a commercially available QD chip from Sparrow Quantum, featuring InAs QDs embedded in a photonic crystal waveguide that emit within the wavelength range of 920–940 nm [45], the measured transmission of the waveguide is around 32 %. The chip is mounted on a three-axis piezo stage and cooled to 5 K in a helium-bath cryostat. A pulsed ps Ti:Sapphire laser (Coherent MIRA HP-P, 76 MHz rep. rate, ≈2.5 ps pulse duration) resonantly excites the QD through a 4*f*-filter that narrows the spectral bandwidth to approximately 0.05 nm and correspondingly extends the pulse duration to ≈25 ps.

An aspheric lens (Thorlabs C340TMD-B) with a numerical aperture of 0.64 is used for both focusing the excitation laser onto the QD and collecting the QD emission. To separate the excitation and collection paths, a 90:10 (R:T) beam splitter is employed, while laser rejection is achieved through cross-polarized excitation/collection using a polarizing beam splitter (PBS) with an extinction ratio of 500:1. The QD emission is filtered using an etalon (Light Machinery, OP-3644-1000). Finally, the filtered light is coupled into a polarization-maintaining (PM) fiber with coupling efficiency of 55 %, and the polarization was aligned to the slow-axis of the PM fiber via a half-wave plate (HWP). The output end of the fiber is connected to an EOPM (Exail NIR-800-20L, 55 % transmission at 930 nm), which implements the electro-optic frequency translator, described in the next section.

### 2.2 Electro-optic frequency translator

We implement an electro-optic frequency translator by driving an EOPM with a radio-frequency (RF) sawtooth waveform. This results in application of an effective linearly time-varying phase to the single-photon wavepackets, which shifts their frequency without changing their temporal shape. The process can be described by applying a temporal phase modulation $e^{\pm i\left(2\pi f_{m}t\right)\mathrm{mod}\left(2n\pi \right)}$ , where *f*_*m*_ is the modulation frequency. Such temporal phase modulation results in a frequency shift of Δ*f* = ±*nf*_*m*_. When *n* is an integer, this is called serrodyne modulation [43], which ideally shifts the frequency without distorting the photon’s temporal shape.

However, generating a high-frequency serrodyne signal is experimentally difficult due to the required RF power and bandwidth [20]. In our case, the available RF power is about 22 dBm, and the modulator’s half-wave voltage is around 4.5 V. This limits us to approximate serrodyne modulation, where the phase modulation amplitude is less than 2*π* and the phase exhibits discontinuous jumps.

When such a modulation is applied to photons of temporal duration extending to a few periods of the serrodyne, partial frequency shifts occur for each RF period encountered by the photons, leading to sidebands in the spectrum. Fortunately, for photons with an exponentially decaying temporal shape, such as those emitted by QDs, the distortions caused by this modulation are minimal, because the amplitudes of subsequent sidebands are exponentially smaller. When using lower modulation frequencies, i.e., longer saw-tooth periods, the photon wavepacket encounters fewer periods; hence, smaller distortions occur, as confirmed by numerical simulation.

In our experiment, we generate a sawtooth waveform with a repetition frequency of 4.56 GHz, using an arbitrary waveform generator (AWG, Keysight M8196A) with a 35 GHz RF bandwidth. The RF waveform is amplified using a 50 GHz broadband amplifier (Keysight N4985A S50). This results in spectral shifts of ±(3.5 ± 0.4) GHz, ±(0.010 ± 0.001) nm, as discussed further in the next section, where we also describe the setups used to characterize and compare the shifted and unshifted photons.

### 2.3 Measurements and source characterization setup

We employed three measurement schemes to characterize the QD source and the performance of the electro-optic frequency translator.

(i) Spectral measurements: To measure the spectrum of the QD emission and the frequency-shifted photons, the output of the EOPM was routed to a grating spectrometer (port 1 in Figure 1), and the spectrometer is composed of a monochromator (Acton SP-2750i) and a CCD camera (Andor Newton). The spectral resolution of the spectrometer is around 15 GHz. The spectral measurements were used to quantify the magnitude of the frequency shift and verify the quality of the shifted spectra.
(ii) Single-photon purity: The purity of the single-photon source was measured using a Hanbury-Brown and Twiss (HBT) setup. The output of the EOPM was directed to port 2, i.e., input of the second fiber beam splitter (bypassing the setup highlighted in the black dashed box in Figure 1). The output of the beam splitter is connected to two fiber-coupled APDs (Perkin Elmer, SPCM-AQRH-14-FC), and a time-tagger (Hydra Harp 400 M) was used for coincidence measurements to obtain the second-order correlation function *g* ^(2)^(*τ*).
(iii) Single-photon indistinguishability: To measure the indistinguishability of consecutive photons, the output of the EOPM was directed to an unbalanced in-fiber Mach–Zehnder interferometer (port 3 in Figure 1), with a 26 ns delay in one arm and a tunable delay line in the other arm. The transmission of the interferometer was around 1.7 %, primarily due to fiber bending losses caused by tight space constraints. Time-tagged avalanche photodetectors (APDs) were used to record coincidence events, allowing us to perform a Hong–Ou–Mandel (HOM) experiment. We perform HOM interference with unshifted photons (EOPM off), blue-shifted, and red-shifted photons. The outcomes are discussed in the next section.

In all cases, when no voltage was applied, the EOPM was passive, enabling comparisons between shifted and unshifted photons. Additionally, multiple phase modulation frequencies were used to demonstrate the tunability of the spectral shift.

## 3 Results and discussion

This section presents the results of our electro-optic frequency translation experiment, including the measured spectral shifts, single-photon purity, and photon indistinguishability. These measurements demonstrate that the electro-optic phase modulation preserves the key qualities of single-photon emission, even after shifting their frequency.

**Figure 2:**
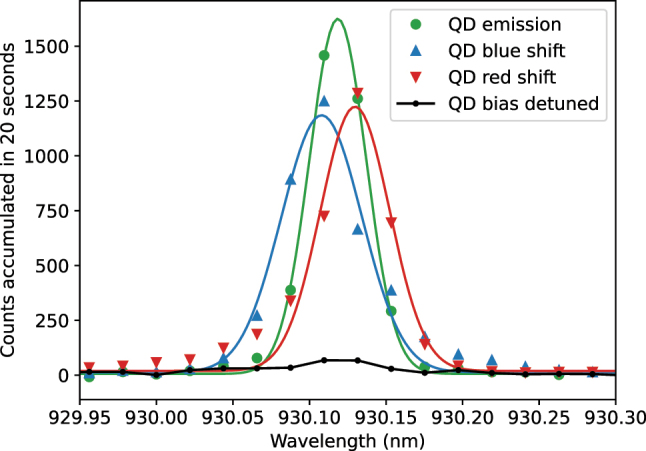
Spectra of the QD emission. The green trace shows the emission spectrum of the QD under pulsed-resonant excitation, while the black trace shows the background with detuned QD bias. The blue and red traces show the electro-optically blue-shifted and red-shifted spectra, respectively. A clear spectral shift of approximately 3.5 GHz (0.01 nm) is observed when the electro-optic phase modulator (EOPM) is activated. Solid-lines are Gaussian fits to the data. A background spectrum with the laser off is subtracted from all the spectra.

The emission spectrum of the QD under pulsed resonant excitation is shown in Figure 2. By adjusting the waveplates in the collection path, we minimized the amount of leaked laser signal, resulting in a signal-to-noise ratio (SNR) of approximately 21, which is limited by the extinction ratio of the polarization controls.

In the experiment, we applied a 4.56 GHz sawtooth waveform generated by a 35 GHz bandwidth AWG to the EOPM. By changing the sign of the slope of the sawtooth waveform, this setup produced both red and blue spectral shifts. The red and blue spectral shifts are clearly visible in Figure 2, which shows traces of the QD emission spectra before and after the modulation. By fitting the spectral data with a Gaussian function, we measured a frequency shift of ±3.5 ± 0.4 GHz, corresponding to a wavelength shift of ±0.010 ± 0.001 nm. The uncertainty is derived from error propagation in the fitting parameters. The shift’s magnitude corresponds to approximately 3.5 times the linewidth of Γ ≈1 GHz, which is a typical value of the inhomogeneous broadening observed for this type of nanostructures [45].

**Figure 3:**
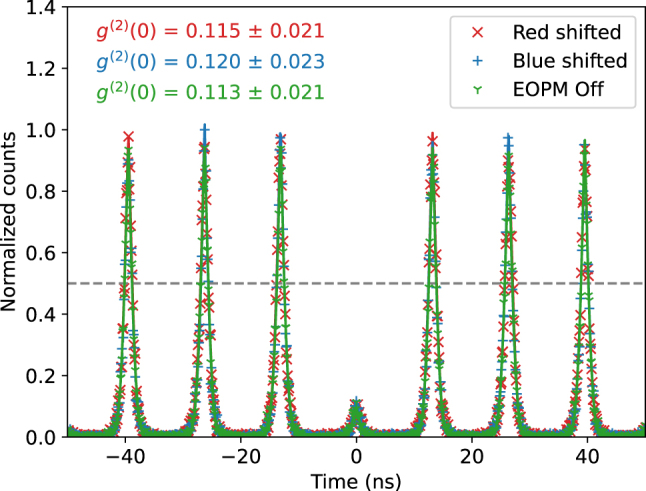
Second-order correlation measurements of the single-photon source. The green trace shows the correlation histogram when the electro-optic phase modulator (EOPM) is off, indicating a photon purity of 88.7 % ± 2.1 %. The red and blue traces represent the results for red-shifted and blue-shifted photons, respectively, with a similar *g* ^(2)^(0) values within the error bars, confirming that the frequency shift does not degrade the single-photon purity.

The purity of the single-photon source was assessed using a Hanbury-Brown and Twiss (HBT) setup, which allowed us to measure the second-order correlation function at zero delay, *g* ^(2)^(0). The measured correlation histograms are shown in Figure 3.

When the EOPM was off (green trace), we obtained a *g* ^(2)^(0) value of 0.113 ± 0.021. Idealy, *g* ^(2)^(0) should be zero for a true single-photon source. However, the measured value indicates some multiphoton contribution, which we predominantly attribute to the leak from excitation laser. Nonetheless, for both the red-shifted and blue-shifted photons, the measured *g* ^(2)^(0) values remained within uncertainty limits, indicating that the frequency shifting process did not degrade the purity of the single-photon source.

**Figure 4:**
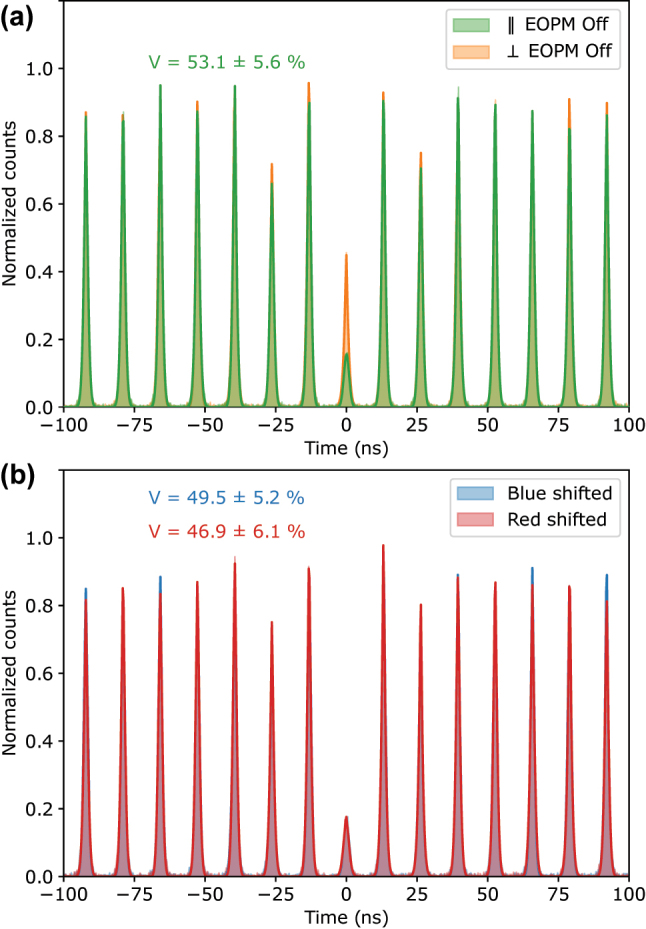
Photon indistinguishability: Hong–Ou–Mandel (HOM) interference. (a) HOM interference visibility of 53.1 % ± 5.6 % is observed for copolarized (green trace) and cross-polarized (orange trace) consecutive photons from the QD when the electro-optic phase modulator (EOPM) is off. (b) HOM visibility of 49.5 % ± 5.2 % and 46.9 % ± 6.1 % is observed for blue-shifted and red-shifted photons, respectively. These results confirm that the frequency shift does not degrade photon indistinguishability, with small differences falling within experimental uncertainty. The counts were accumulated for 15 min in all measurements.

To assess the indistinguishability of consecutively frequency shifted photons and compare it with the unshifted case, we performed Hong–Ou–Mandel (HOM) interference experiments using an unbalanced in-fiber Mach–Zehnder interferometer. The results, presented in Figure 4, show the normalized coincidence counts for both copolarized and cross-polarized configurations.

The raw visibility from the data is calculated using the following expression:

(1) $$V_{\text{raw}}=1-\frac{A_{\|}}{A_{⊥}},$$

where *A*_‖_ and *A*_⊥_ are the normalized coincidence counts integrated over the central peaks for co- and cross-polarization configuration, respectively.

In the case when the EOPM was off, we measured a raw visibility of 53.1 % ± 5.6 %, and the moderate visibility is likely due to imperfect polarization alignment in the HOM setup and the residual leakage of the excitation laser. For the blue-shifted and red-shifted photons, the raw visibilities were 49.5 % ± 5.2 % and 46.9 % ± 6.1 %, respectively. These values suggest that the frequency shift introduced by the EOPM does not significantly affect photon indistinguishability, as the small variations in visibility are within the experimental uncertainties. The subsequent degradation in visibility from 53.1 % to 49.5 % to 46.9 % is consistent with the order of the measurements, which can be attributed to the slow drifts in stability of the experimental setup. It would be interesting to perform the experiment with higher visibility to better understand the degradation in visibility due to the timing jitter of the RF signal and other sources of noise, and also exploring the excitation pulse area effects on the photon number statistics.

Similar setups have demonstrated visibilities greater than 90 % under optimized conditions, such as with higher-quality polarizers [13], [14]. One can improve the visibility by better filtration of the laser; however, the goal of this work is not to demonstrate high visibility HOM interference but rather to show that the electro-optic frequency shift does not introduce significant degradation in photon indistinguishability.

**Figure 5:**
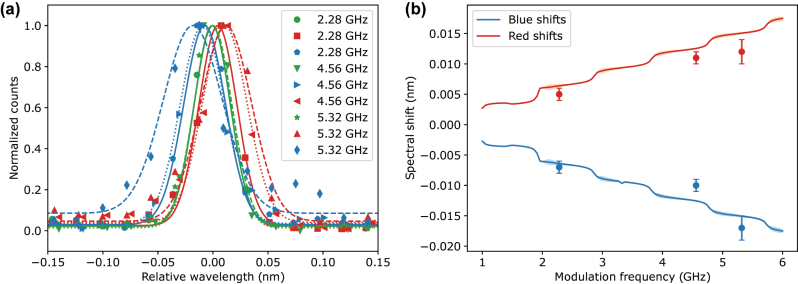
Tunable spectral shift using different modulation frequencies of serrodyne signal. (a) Normalized emission spectra of the QD for three different modulation frequencies 2.28 GHz (solid lines), 4.56 GHz (dotted lines), and 5.32 GHz (dashed lines). The blue and red shifts are achieved by changing the sign of the slope of serrodyne modulation. The magnitude of the spectral shift increases monotonically with the modulation frequency. The lines represent Gaussian fits to the data. (b) Plot of the measured spectral shift as a function of modulation frequency, showing good agreement with realistic simulations. The solid lines are derived from simulations of serrodyne electro-optic shift. Parameters of the modulation were estimated from specifications of the amplifier and the modulator. Frequency response of the modulator and finite bandwidth of the AWG were incorporated to make simulations experimentally realistic.

In addition to the results for the 4.56 GHz modulation, we also demonstrated the tunability of the spectral shift by varying the modulation frequency applied to the EOPM. Figure 5(a) shows the normalized QD emission spectra for serrodyne repetition frequencies of 2.28 GHz, 4.56 GHz, and 5.32 GHz. As expected, the magnitude of the spectral shift increased consistently with the modulation frequency. In particular, the electro-optic frequency shift can be tuned from zero [46].

The spectral shifts obtained from these measurements, shown in Figure 5(b), align well with simulations of the serrodyne frequency translation process. These simulations are based on the specifications of the amplifier and modulator, and they also account for the finite bandwidth of the AWG and the modulator’s frequency response (following the approach of Ref. [47]).

For the simulations, the lifetime of the QD is extracted by fitting the *g* ^(2)^(*τ*) measurements with an exponential function convolved with a Gaussian. The obtained QD lifetime is 1.30 ns ± 0.17 ns.

Our results demonstrate that serrodyne electro-optic phase modulation can achieve a deterministic frequency shift of single photons from a QD source, without compromising the purity or indistinguishability of the photons. The magnitude of the spectral shift in our experiment was limited by the RF power applied to the EOPM and the design wavelength of the modulator. In principle, larger shifts could be achieved with higher-power RF amplifiers and EOPMs optimized for the QD emission wavelength (930 nm). For example, on a thin-film lithium niobate platform with half-wave voltage smaller than 1 V, spectral shifts of ±60 GHz can be achieved by driving the modulator with a 15 GHz serrodyne of order 4 [31], [48], [49]. Stable and tunable frequency shifts, as demonstrated here, are critical for integrating remote QDs into large-scale quantum networks or interfacing them with quantum memories that operate at different wavelengths. For example, the ability to tune GaAs QDs emitting around 780 nm [41], [50], to match the D2 transition of rubidium atoms (780 nm), could enable efficient photon storage in Rb-based quantum memories [51]. Moreover, the electro-optic approach in combination with integrated photonics is a potentially promising approach toward efficient scaling in the context of quantum networks [8], [9].

## 4 Conclusion and outlook

In summary, we have demonstrated that the spectrum of single photons emitted from a QD can be deterministically shifted using an electro-optic phase modulator. We experimentally confirm that the spectrally shifted photons inherit the purity and indistinguishability of the unshifted photons. Electro-optic phase modulation is deterministic [20], i.e., all photons coming out of the phase modulator are spectrally modified, unlike nonlinear frequency mixing, where unconverted photons can be present at the output, which are hard to filter. Also, the phase modulators are available for various wavelengths from visible to mid-infrared [44], [52]. Using multiple phase modulators one can interfere photons from remote QDs to form an *n*-photon state. Whereas free-space implementation of the approach is challenging, due to very high voltages required for driving bulk electro-optic phase modulators and their slow electronic response, the recent integration of QD with a thin-film lithium niobate platform makes on-chip electro-optic phase modulation possible [8], [9]. Efficient aribitrary electro-optic spectral-temporal shaping QD single-photon wave packets is an interesting future research question [20]. Additionally, direct frequency shifting enables frequency-bin encoding on QD photon for quantum key distribution applications [53], [54], [55], [56].
